# Pulmonary Artery Fibroelastoma Excision in a 67-Year-Old Female Patient Presented With a Chronic Cough

**DOI:** 10.1016/j.jaccas.2025.105276

**Published:** 2025-10-29

**Authors:** Raisa Bushra, Jade Alice Loughran, Amit Ranjan, Daniel Fudulu

**Affiliations:** a Cardiothoracic Surgery, Bristol Heart Institute, Bristol, United Kingdom; b Cardiac Intensive Care Unit, Bristol Heart Institute, Bristol, United Kingdom; c Consultant Cardiac Anesthetist and Cardiac Intensive Care Unit, Bristol Heart Institute, Bristol, United Kingdom; d Consultant Senior Lecturer in Cardiac Surgery, Bristol Heart Institute, Bristol, United Kingdom

**Keywords:** cardiac tumor, echocardiography, papillary fibroelastoma, pulmonary valve, surgical excision

## Abstract

**Background:**

Papillary fibroelastoma is a rare, benign cardiac tumor, most commonly found on left-sided heart valves. Right-sided involvement, particularly the pulmonary valve, is exceedingly rare but carries a high embolic risk.

**Case Summary:**

A 67-year-old woman presented with chronic cough and dyspnea. Imaging revealed a mobile pulmonary valve mass, initially suspected as vegetation. Further evaluation with echocardiography and magnetic resonance imaging suggested fibroelastoma. Due to diagnostic challenge and high embolic risk, a diagnostic surgical excision was planned.

**Discussion:**

This case highlights diagnostic challenges and reinforces the importance of considering cardiac tumors in atypical presentations.

## Graphical Abstract

**Figure:**
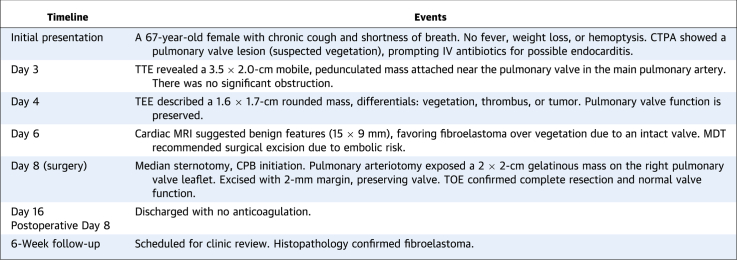

## History of Presentation

**Figure 1:**
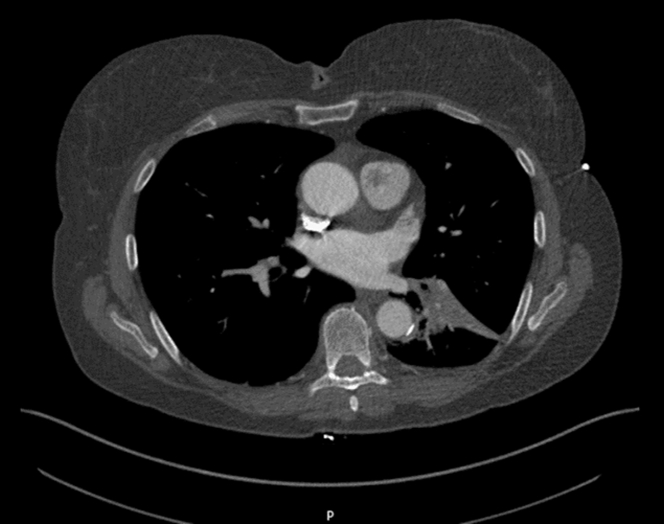
Computed Tomography Pulmonary Angiogram Showing Filling Defect in the Pulmonary Artery

A 67-year-old, nonsmoker, female with a background of meningioma on 5-year surveillance presented with a chronic cough and shortness of breath on exertion. There was no history of hemoptysis, fever, weight loss, or night sweats. On auscultation, bilateral coarse crackles were noted. Raised D-dimer prompted a computed tomography pulmonary angiogram, which showed no evidence of pulmonary embolism but raised concern of a lesion on the pulmonary valve, presumed to be a vegetation (Figure 1). As a result, she was treated with intravenous antibiotics for infective endocarditis. Transthoracic echocardiography was performed to further investigate, which revealed a mobile, pedunculated echogenic mass measuring approximately 3.5 × 2.0 cm in the proximal aspect of the main pulmonary artery. The mass appeared to originate from the pulmonary valve but did not cause significant flow obstruction.

**Figure 2:**
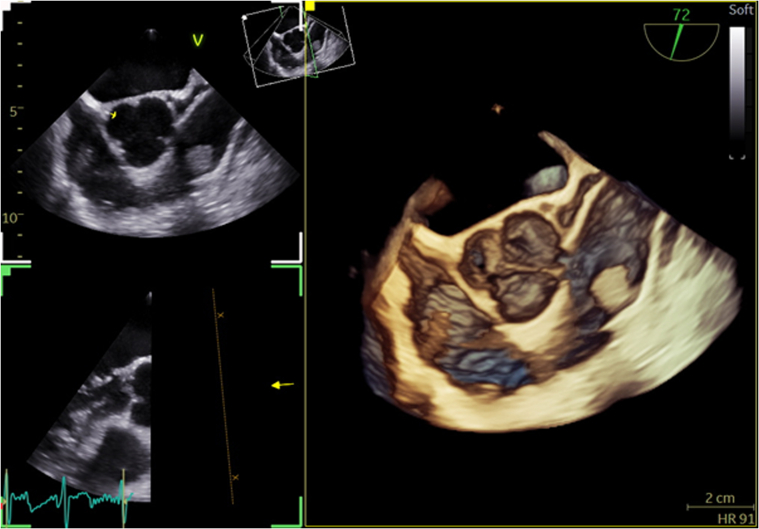
Preoperative Transesophageal Echocardiography 3D Images: Right-Sided Papillary Fibroelastoma

Transesophageal echocardiography was arranged. This was reported as showing a “mobile and fairly rounded pedunculated mass/structure in the main pulmonary artery attached to or near the pulmonary valve anteriorly” (Figure 2). It measures, in some views, 1.6 × 1.7 cm. This appearance was felt to be consistent with vegetation, or other pathology, for example, thrombus or tumor. Pulmonary valve function was preserved, with only trivial pulmonary regurgitation.

Further investigation with magnetic resonance imaging again demonstrated the mass measuring 15 × 9 mm, describing it as pulmonary valve related, with benign features, and noting that the absence of valve destruction made vegetation an unlikely diagnosis, with fibroelastoma being the most likely diagnosis.

The multidisciplinary team discussion and imaging review concluded that surgical excision was warranted due to the high embolic risk and the need for a definitive diagnosis.

Excision of the pulmonary valve tumor via median sternotomy was the planned operation.

**Figure 3:**
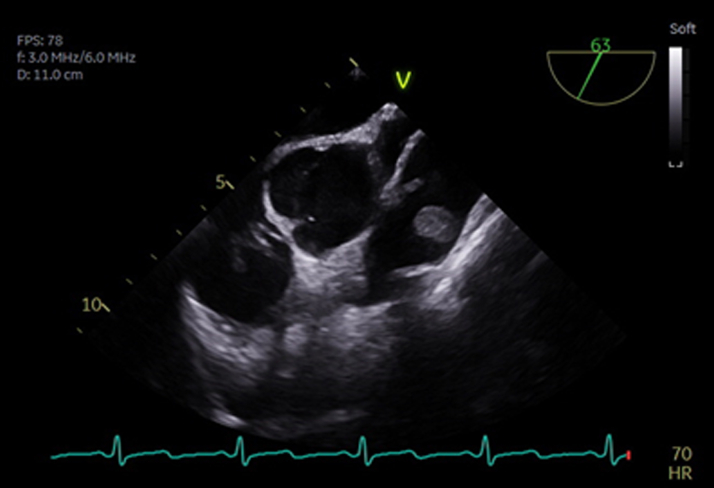
Intraoperative Transesophageal Echocardiography Images: Right-Sided Papillary Fibroelastoma

**Figure 4:**
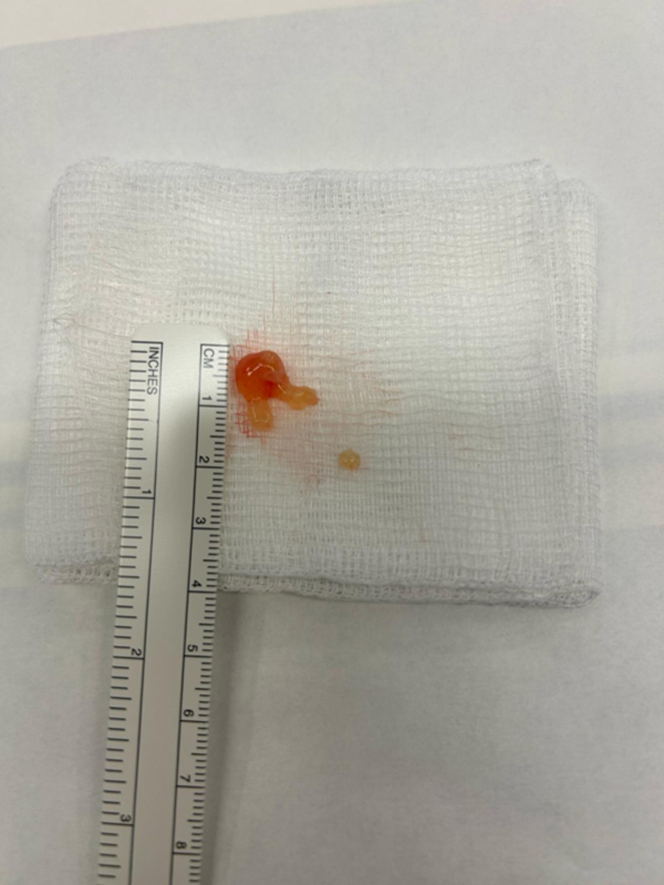
Intraoperative Pictures of the Right-Sided Papillary Fibroelastoma

After median sternotomy, aortic, and bicaval cannulation, cardiopulmonary bypass was initiated. The pulmonary artery was opened longitudinally, exposing the tumor. Intraoperative findings were a gelatinous 2 × 2-cm pink mass with a narrow stalk attached to the right pulmonary valve leaflet (Figures 3 and 4). The valve itself appeared structurally normal.

Thus, the tumor was carefully excised with a 2-mm margin of normal endocardium to ensure complete resection while preserving valve integrity (Figure 4). The arteriotomy was closed in 2 layers.

**Figure 5:**
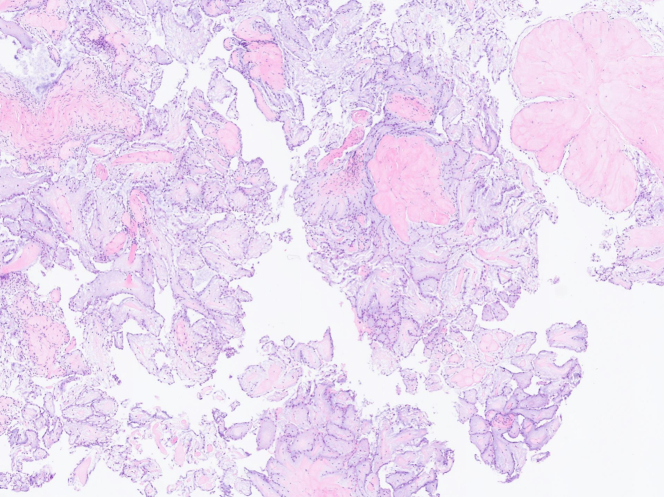
Microscopic Histopathology Picture of Right-Sided Papillary Fibroelastoma

Postresection TOE confirmed no residual mass and normal valve function. Histopathology demonstrated classic fibroelastoma features (Figure 5): avascular fibrous cores lined by benign endothelium, with no malignancy.

The patient had an uncomplicated postoperative recovery. On postoperative day 8, the patient was discharged with no anticoagulation until the histopathology report and scheduled for follow-up in the clinic after 6 weeks.

## Discussion

Papillary fibroelastoma is a rare, benign endocardial tumor that predominantly arises from valvular surfaces, particularly the aortic and mitral valves.^1^ First described in detail by Yater^2^, Papillary fibroelastoma accounts for approximately 10% of all primary cardiac neoplasms, making it the second most common after myxomas.^1^ Histologically, they are characterized by avascular frond-like projections composed of a central core of dense connective tissue surrounded by endothelial cells, giving them a distinctive “sea anemone” appearance on gross examination.^3^

Although most fibroelastomas are found on heart valves, they can also develop in nonvalvular locations, including the left ventricular septum, atrial endocardium, and rarely, the great vessels.^4^ Valvular lesions predominantly affect the left side of the heart (79% of cases),^5^ (Hajouli S, 2024) while right-sided valve lesions are exceptionally uncommon, with only about 20 cases reported in the literature.^6^ Despite their benign histology, their fragile papillary architecture poses a significant thromboembolic risk, potentially leading to stroke, pulmonary embolism, or coronary occlusion.^7^

The clinical presentation of fibroelastoma varies widely, ranging from asymptomatic incidental findings to life-threatening embolic events. Surgical excision remains the definitive treatment.^8^

They may present with nonspecific symptoms such as dyspnea, chest pain, syncope, or the right ventricular outflow obstruction signs, which can mimic more common conditions like pulmonary embolism, pulmonary hypertension, or pulmonary valve stenosis.^9^ Thus, this nonspecific presentation highlights the diagnostic challenge and significance of considering rare cardiac tumors as a differential diagnosis.

The primary concern with fibroelastomas, regardless of location, is their embolic potential. The tumor's papillary architecture and turbulent blood flow in the great vessels increase the risk of thrombus formation and subsequent systemic or pulmonary embolism.^1^

For diagnosis, the first-line imaging tool is transthoracic echocardiography which initially detected the mass. However, more invasive TEE provided superior resolution and view, and more specifically, it confirmed the pedunculated, mobile nature and attachment site of the mass.^10^

Cardiac magnetic resonance imaging is also an excellent imaging tool that offers additional structural characterization. It differentiates the tumor from thrombus or myxoma based on its T2 hyperintensity and frond-like morphology.^11^

Histopathology is the gold standard for diagnosis, revealing the classic endothelial-covered fibroelastic strands pathognomonic for fibroelastoma.

After diagnosis, early intervention is crucial to avoid complications such as right heart failure, distal embolization, or sudden cardiac death.^12^ Surgical excision remains the treatment of choice, either a conventional or minimally invasive procedure, for symptomatic or mobile fibroelastomas to avoid the high risk of embolism.

Fibroelastomas have an excellent prognosis postresection, with negligible recurrence risk.^4^ However, lifelong surveillance echocardiography is recommended to monitor potential complications or recurrence.^13^

## Conclusions

Although rare, right-sided papillary fibroelastoma should be considered a differential diagnosis in patients presenting with unexplained dyspnea or pulmonary artery masses.^14^ A high index of suspicion, combined with multimodal imaging, is essential for accurate diagnosis, a holistic multidisciplinary team approach, and timely intervention.^11^ Surgical excision remains the definitive treatment and is associated with favorable outcomes.^9^

## Funding Support and Author Disclosures

The authors have reported that they have no relationships relevant to the contents of this paper to disclose.

**Take-Home Messages**

• Pulmonary valve fibroelastoma, although rare, should be considered in unexplained pulmonary symptoms.
• Early surgical intervention prevents severe complications and ensures favorable outcomes.

**Visual Summary:** Atypical Presentation of Pulmonary Artery Fibroelastoma

Timeline	Events
Initial presentation	A 67-year-old female with chronic cough and shortness of breath. No fever, weight loss, or hemoptysis. CTPA showed a pulmonary valve lesion (suspected vegetation), prompting IV antibiotics for possible endocarditis.
Day 3	TTE revealed a 3.5 × 2.0-cm mobile, pedunculated mass attached near the pulmonary valve in the main pulmonary artery. There was no significant obstruction.
Day 4	TEE described a 1.6 × 1.7-cm rounded mass, differentials: vegetation, thrombus, or tumor. Pulmonary valve function is preserved.
Day 6	Cardiac MRI suggested benign features (15 × 9 mm), favoring fibroelastoma over vegetation due to an intact valve. MDT recommended surgical excision due to embolic risk.
Day 8 (surgery)	Median sternotomy, CPB initiation. Pulmonary arteriotomy exposed a 2 × 2-cm gelatinous mass on the right pulmonary valve leaflet. Excised with 2-mm margin, preserving valve. TOE confirmed complete resection and normal valve function.
Day 16 Postoperative Day 8	Discharged with no anticoagulation.
6-Week follow-up	Scheduled for clinic review. Histopathology confirmed fibroelastoma.

CPB = cardiopulmonary bypass; CTPA = CT pulmonary angiogram; IV = intravenous; MDT = multidisciplinary team; MRI = magnetic resonance imaging; TEE = transesophageal echocardiography; TTE = transthoracic echocardiography.
